# Global, regional, and national burden of nutritional deficiencies spanning from 1990 to 2021, with a focus on the impacts observed during the COVID-19 pandemic

**DOI:** 10.3389/fnut.2025.1535566

**Published:** 2025-05-08

**Authors:** Yue-Yang Zhang, Bing-Xue Chen, Qin Wan

**Affiliations:** ^1^Department of Endocrinology and Metabolism, Affiliated Hospital of Southwest Medical University, Luzhou, China; ^2^Metabolic Vascular Disease Key Laboratory of Sichuan Province, Luzhou, China; ^3^Sichuan Clinical Research Center for Diabetes and Metabolism, Luzhou, China; ^4^Sichuan Clinical Research Center for Nephropathy, Luzhou, China; ^5^Cardiovascular and Metabolic Diseases Key Laboratory of Luzhou, Luzhou, China; ^6^Department of Ultrasound Medicine, Affiliated Hospital of Southwest Medical University, Luzhou, China

**Keywords:** nutritional deficiencies, global burden, dietary iron deficiency, vitamin A deficiency, protein-energy malnutrition, iodine deficiency

## Abstract

**Background:**

The United Nations has recognized nutritional deficiencies as a critical health issue that necessitates urgent eradication. This study aimed to provide a comprehensive analysis of the spatial distribution and temporal trends of the global disease burden associated with nutritional deficiencies and their four subtypes from 1990 to 2021, with a particular focus on the impact of the COVID-19 pandemic.

**Methods:**

This study primarily employs the most recent data from the Global Burden of Disease (GBD) 2021 to conduct a thorough analysis of the distribution trends of incidence, mortality, and disability-adjusted life years (DALYs) associated with nutritional deficiencies and their four subtypes from 1990 to 2021, incorporating detailed subgroup analyses categorized by sex, age, and region. In comparison to the GBD 2019, the GBD 2021 update places a particular emphasis on supplementing disease burden data for the period of the COVID-19 pandemic (2019–2021). Furthermore, this study investigates the primary risk factors contributing to disability-adjusted life years (DALYs) linked to nutritional deficiencies.

**Results:**

Between 1990 and 2021, the global burden of nutritional deficiencies experienced a substantial decline, evidenced by a 54.9% reduction in the age-standardized incidence rate (ASIR), a 72.2% decrease in the age-standardized death rate (ASDR), and a 51.9% reduction in the age-standardized DALY rate. However, it is noteworthy that the burden of iodine deficiency (ASIR: 137.72 vs. 75.49; Age-standardized DALY rate: 35.43 vs. 19.98) and dietary iron deficiency (Age-standardized DALY rate: 597.97 vs. 253.05) is considerably greater in women than in men. Moreover, in regions characterized by a low social demographic index (SDI) and lower income levels, the burden of diseases associated with nutritional deficiencies remains substantial. In contrast, the COVID-19 pandemic has not markedly changed the epidemiological profile of nutritional deficiencies compared to the pre-2019 period, and the global burden of nutritional deficiencies has continued its gradual decline.

**Conclusions:**

Despite a decline in the global burden of nutritional deficiencies over time, significant disparities related to gender, region, and age persist. Fortunately, the COVID-19 pandemic has had a relatively limited impact on the global burden of nutritional deficiencies. Healthcare institutions must formulate more targeted strategies aimed at alleviating the adverse effects of nutritional deficiencies on global public health.

## Introduction

Nutritional deficiencies are typically defined as a pathological state resulting from inadequate intake, poor absorption, or excessive loss of essential nutrients (including vitamins, minerals, and proteins); chronic malnutrition can result in multi-system damage, encompassing neurological, endocrine, and cardiovascular complications, and may ultimately lead to death (1–4). A report from the World Health Organization (WHO) indicates that nutritional deficiencies are widespread in both developed and developing countries, significantly affecting public health, particularly among vulnerable populations, including children, pregnant women, and the elderly (5, 6). The epidemiological profile of nutritional deficiencies highlights the intricate interactions among various risk factors. The 2021 Global Nutrition Report indicates that nearly 2 billion adults are classified as overweight or obese, yet one in three adults experiences at least one form of micronutrient deficiency. Among children, stunting and wasting continue to pose significant challenges, especially in low- and middle-income countries (7, 8). Consequently, the United Nations General Assembly proclaimed the period from 2016 to 2025 as the United Nations Decade of Action on Nutrition, with the objective of eradicating hunger and preventing all forms of malnutrition (9).

Nutritional deficiencies are typically classified into two primary categories: macronutrient deficiencies and micronutrient deficiencies (10). Macronutrient deficiencies specifically pertain to inadequate intake of proteins, carbohydrates, and fats (11). Among these, protein-energy malnutrition represents a critical concern in numerous low-income countries, often resulting in diseases such as kwashiorkor and marasmus (12, 13). Micronutrient deficiencies primarily denote a lack of essential vitamins and minerals; although the human requirement for these nutrients is relatively minimal, they play a vital role in various physiological functions (14). For instance, iodine deficiency may result in goiter and cognitive impairment, particularly among children (15). Vitamin A deficiency can adversely impact vision and immune function, and it is recognized as one of the leading causes of blindness (16).

Existing research indicates that the global burden of nutritional deficiencies remains prevalent as of 2019, highlighting the need for focused attention on the more pronounced burden in economically disadvantaged regions due to disparities in economic development (17). Unfortunately, it is well established that the COVID-19 pandemic has rendered the global nutritional landscape more complex, disrupting existing food supply chains and exacerbating disparities in nutritional health (18, 19). However, the extent to which the COVID-19 pandemic has influenced the global burden of nutritional deficiencies and whether it has altered the pre-existing epidemiological patterns remains unclear. No studies have yet reported changes in the global burden of nutritional deficiencies during the COVID-19 pandemic. To address this gap, this study utilizes the most recent Global Burden of Disease (GBD) 2021 data to evaluate the burden of nutritional deficiencies across 204 countries and territories worldwide from 1990 to 2021, thereby expanding on previous research and filling the data void caused by the pandemic of COVID-19. Furthermore, this study examines the impact of various risk factors on disability-adjusted life years (DALYs) associated with nutritional deficiencies. Compared to previous GBD studies, GBD 2021 represents a significant advancement by incorporating updated data from the COVID-19 pandemic, enabling researchers to assess epidemiological trends and explore the potential impact of COVID-19 on disease burden.

## Method

### Data source

The GBD 2021 comprehensively evaluated over 370 diseases and injuries across 204 countries and regions globally, delivering detailed data on incidence, death, DALYs, and their corresponding age-standardized rates (20, 21). All data were derived from publicly available databases established by national institutions, with numerous published studies integrated and subjected to rigorous quality control measures to ensure data accuracy and reliability (22, 23).

### Data definitions

Total incidence and age-standardized incidence rates (ASIR) were evaluated using Bayesian meta-regression models, whereas total mortality and age-standardized mortality rates (ASDR) were predominantly estimated utilizing the Cause of Death Ensemble Model (CODEm). Disability weights quantify the severity of health loss or non-fatal disabilities, while DALYs reflect the total years lost due to health impairment, from the onset of disease to death, serving as a crucial indicator for evaluating disease burden (24, 25).

The Socio-Demographic Index (SDI) is a composite measure that reflects a country's level of development, incorporating per capita income, the average education level of individuals aged 15 and older, and total fertility rates among individuals under 25 years. This index is closely associated with health outcomes, with SDI values ranging from 0 to 1, where 0 signifies the lowest level of development and 1 denotes the highest level of development (26, 27).

Based on SDI values, the 204 countries and regions were categorized into five distinct groups: high SDI, medium-high SDI, medium SDI, medium-low SDI, and low SDI regions. Patients were stratified into twelve age groups: <5 years, 5–9 years, 10–14 years, 15–19 years, 20–24 years, 25–29 years, 30–34 years, 35–39 years, 40–44 years, 45–49 years, 50–69 years, and 70+ years.

### Definition of nutritional deficiencies

In the GBD 2021 study, the definition of nutritional deficiencies is grounded in the codes for nutritional deficiencies outlined in the 10th edition of the International Classification of Diseases (ICD), encompassing protein-energy malnutrition (ICD-10 codes E40–E46.9, E64.0), iodine deficiency (E00–E02), vitamin A deficiency (E50–E50.9, E64.1), and dietary iron deficiency (D50–D50.9) (28).

### Estimation of risk factor

We assessed 70 specific attributable risk factors identified in GBD 2021, including particulate pollution, extreme temperatures, lead exposure, smoking, secondhand smoke exposure, high consumption of red meat and sodium, low intake of fiber, fruits, and vegetables, as well as elevated fasting blood glucose levels. However, owing to limitations in data availability, the final attribution analysis predominantly concentrated on iron deficiency, vitamin A deficiency, child underweight, and child wasting.

### Patient and public involvement

It was not appropriate or possible to involve patients or the public in the design, or conduct, or reporting, or dissemination plans of our research.

### Statistical analyses

We evaluated the global burden of nutritional deficiencies by analyzing annual incidence, total death, DALYs, and the corresponding ASRs. Age-standardized rates serve to mitigate the effects of disparate age distributions and demographic shifts across various regions, thereby ensuring the comparability of study metrics. Utilizing the ASIR, ASDR, and age-standardized DALYs, we calculated estimates of the annual percentage change (EAPC) to retrospectively analyze the trends in the burden of nutritional deficiencies over the past 32 years. In the equation y = α + βx, y denotes the log10 (ASR) value, while x signifies the year. The formula for calculating EAPC is given by EAPC = 100 ^*^ (10^β^ – 1). If the EAPC value and its 95% confidence interval (CI) exceed zero, this indicates an increasing trend in ASR; conversely, it signifies a decreasing trend (23).

Furthermore, to investigate the correlation between ASIR, ASDR, age-standardized DALY rates, and levels of social development, we computed the Pearson correlation coefficients alongside the SDI values. Statistical analyses were performed utilizing GraphPad Prism (version 10.0.0 for Windows), encompassing *t-*tests and analysis of variance (ANOVA) to compare incidence rates and disability-adjusted life years across diverse genders, age groups, and regions.

All hypothesis tests conducted in this study were two-tailed, with a significance threshold established at *P* < 0.05 (29).

## Result

### Global trends in the burden of nutritional deficiencies

**Figure 1:**
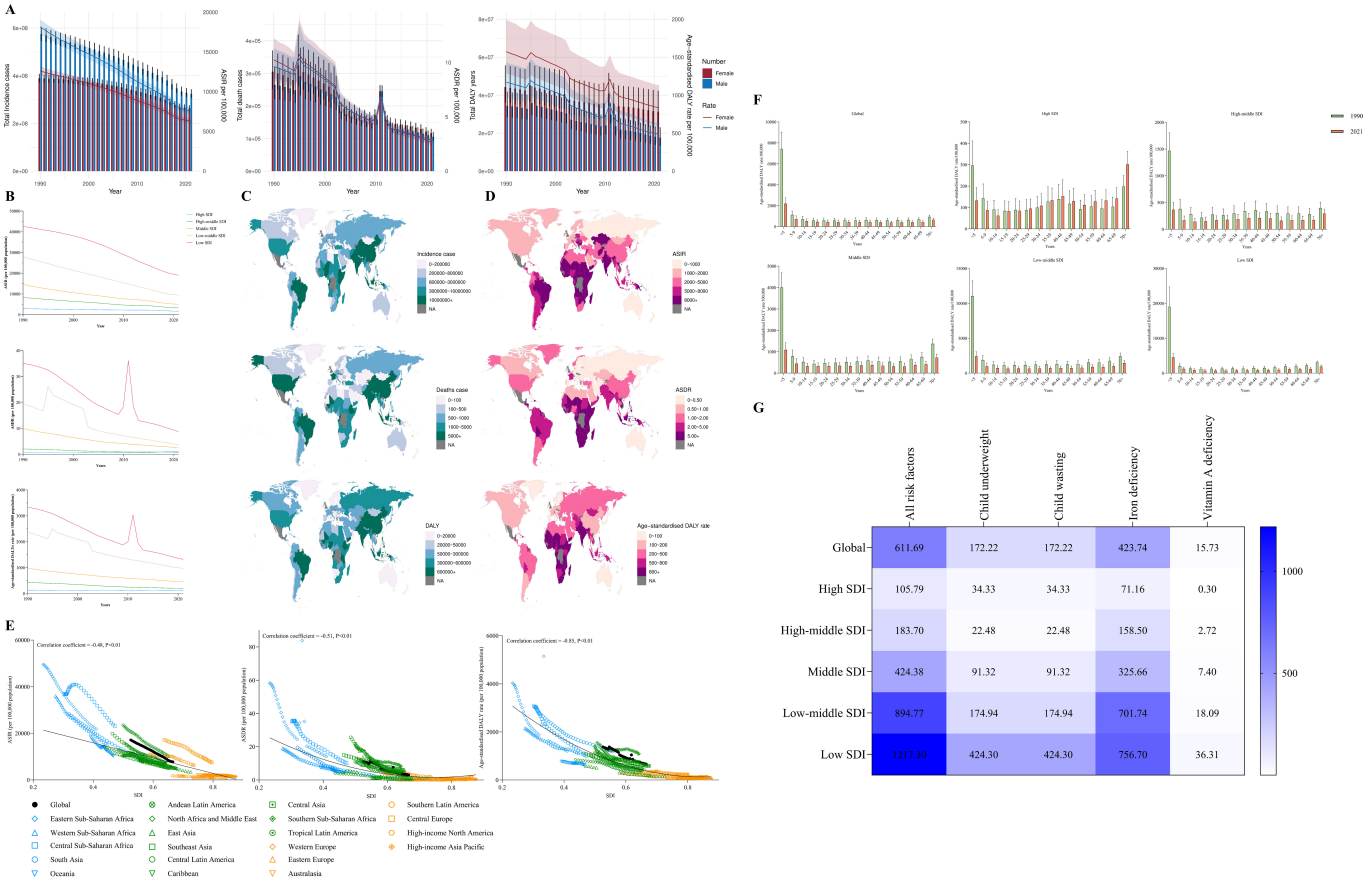
**(A)** The change trends of nutritional deficiencies' incidence cases, death cases and DALY from 1990 to 2021. Blue bars represent males and orange bars represent females. **(B)** Trends from 1990 to 2021 in the ASIR, ASDR and Age-standardized DALY rate of nutritional deficiencies in five SDI regions. **(C, D)** The global disease burden of nutritional deficiencies in 204 countries or territories. **(E)** The change trends and correlation analyses of ASIR, ASDR and Age-standardized DALY rate with SDI from 1990 to 2021. **(F)** The Age-standardized DALY rate of nutritional deficiencies in different age groups in 1990 and 2021. **(G)** Risk factors contributing to nutritional deficiencies -related DALY.

From 1990 to 2021, the global burden of nutritional deficiencies exhibited a consistent decreasing trend. The ASIR decreased by 54.9% (from 17,112.55 per 100,000 in 1990 to 7,725.10 per 100,000 in 2021, EAPC = −2.52), the ASDR declined by 72.2% (from 10.90 per 100,000 in 1990 to 3.03 per 100,000 in 2021, EAPC = −4.41), and the age-standardized DALY rate decreased by 51.9% (from 1,367.15 per 100,000 in 1990 to 657.62 per 100,000 in 2021, EAPC = −2.52). Furthermore, the ASIR for males was generally higher than that for females, whereas the age-standardized DALY rate was elevated for females; however, the difference in ASDR between genders was not statistically significant (Table 1, Figure 1A).

**Table 1:** The incidence of nutritional deficiencies in 1990/2021.

**Variables**	**1990**			**2021**		
	**ASIR/100,000 No. (95% UI)**	**ASDR/100,000 No. (95% UI)**	**Age-standardized DALY rate/100,000 No. (95% UI)**	**ASIR/100,000 No. (95% UI)**	**ASDR/100,000 No. (95% UI)**	**Age-standardized DALY rate/100, 000 No. (95% UI)**
Overall	17,112.55 (16,470.31, 17,731.43)	10.90 (9.44, 12.97)	1,367.15 (1,126.30, 1,708.49)	7,725.10 (7,404.01, 8,109.01)	3.03 (2.69, 3.40)	657.62 (489.93, 869.58)
EAPC-ASIR		−2.52 (−2.67, −2.38)				
EAPC-ASDR		−4.41 (−4.84, −3.98)				
EAPC-Age-standardized DALY rate		−2.52 (−2.71, −2.32)				
**Sex**						
Male	20,170.14 (19,158.18, 21,173.10)	10.71 (9.28, 12.56)	1,171.63 (967.32, 1,430.87)	8,423.99 (7,987.09, 8,934.09)	3.16 (2.80, 3.58)	484.62 (372.00, 631.78)
Female	14,000.41 (13,368.81, 14,655.15)	11.40 (9.51, 13.61)	1,573.03 (1282.74, 1,994.48)	7,006.41 (6,700.12, 7,383.65)	2.96 (2.63, 3.33)	834.92 (598.63, 1,127.32)
**SDI**						
High SDI	2,909.50 (3,180.88, 2,668.01)	0.78 (0.70, 0.84)	131.07 (92.53, 189.66)	1,671.71 (1,484.68, 1,885.64)	0.95 (0.80, 1.03)	118.26 (83.05, 165.11)
High-middle SDI	8,339.40 (7,781.30, 8,950.07)	2.06 (1.86, 2.26)	429.22 (326.50, 575.07)	3,305.31 (3,046.36, 3,589.94)	0.8 (0.7, 0.89)	203.41 (140.77, 288.96)
Middle SDI	14,567.32 (13,862.76, 15,328.32)	9.89 (9.09, 10.56)	972.77 (797.32, 1,229.51)	5,016.29 (4,705.26, 5,381.24)	2.62 (2.34, 2.82)	459.47 (331.59, 624.72)
Low-middle SDI	27,944.93 (26,526.37, 29,160.78)	19.60 (16.61, 22.99)	2,374.12 (1,965.67, 2,980.94)	9,389.32 (8,870.92, 9,951.09)	3.73 (3.33, 4.12)	971.02 (706.71, 1,308.72)
Low SDI	35.05 (28.92, 43.14)	35.05 (28.92, 43.14)	334.19 (2,727.78, 4,117.71)	19,047.59 (18,448.14, 19,697.06)	8.73 (7.44, 10.02)	1,319.28 (1,002.38, 1,722.91)
**Region**						
Central Asia	8,894.18 (8,339.60, 9,462.22)	1.23 (1.13, 1.33)	845.24 (595.7, 1,176.90)	4,927.20 (4,597.18, 5,281.99)	0.32 (0.28, 0.36)	585.12 (399.86, 837.14)
East Asia	11,535.28 (10,239.38, 12,956.89)	5.45 (4.78, 6.10)	570.83 (462.24, 725.04)	3,450.57 (3,075.48, 3,859.46)	1.13 (0.93, 1.32)	160.76 (110.46, 228.34)
South Asia	28,292.51 (26,126.08, 30,300.72)	19.49 (16.02, 23.18)	2,796.88 (2,246.58, 3,574.34)	9,171.97 (8,326.93, 10,138.25)	2.56 (2.16, 3.00)	1,187.87 (840.68, 1,627.64)
Southeast Asia	19,895.08 (18,775.33, 21,118.15)	15.04 (12.61, 17.05)	1,104.99 (899.41, 1,380.21)	5,892.56 (5,501.48, 6,347.38)	5.96 (5.10, 6.65)	505.55 (384.30, 664.82)
Central Europe	17,286.89 (16,504.76, 18,203.53)	0.17 (0.15, 0.18)	360.34 (237.09, 521.60)	7,479.70 (7,086.41, 7,915.69)	0.36 (0.33, 0.39)	200.85 (134.94, 293.55)
Eastern Europe	1,891.24 (1,660.15, 2,160.48)	0.48 (0.46, 0.50)	352.98 (242.19, 508.14)	1,146.35 (947.99, 1,355.48)	0.29 (0.27, 0.31)	236.53 (163.10, 337.20)
Sub-Saharan Africa	38,774.88 (38,136.38, 39,441.60)	33.92 (28.39, 41.91)	2,917.80 (2,401.44, 3,613.64)	18,022.35 (17,539.945, 18,491.50)	9.56 (8.11, 11.03)	1,120.50 (871.92, 1,444.32)
Oceania	18,443.43 (17,325.88, 19,591.87)	7.58 (6.30, 9.10)	776.54 (583.49, 1,037.62)	10,329.04 (9,522.33, 11,202.87)	4.36 (3.57, 5.42)	610.45 (433.75, 897.37)
Latin America and Caribbean	16,808.45 (15,883.74, 17,764.75)	17.61 (16.72, 18.30)	1,121.27 (980.98, 1,319.18)	7,346.28 (6,882.37, 7,924.83)	3.94 (3.51, 4.37)	399.33 (305.24, 515.72)
**World Bank Income Level**						
World Bank High Income	3,638.71 (3,401.69, 3,912.01)	0.77 (0.70, 0.83)	142.22 (99.89, 203.76)	1,960.85 (1,778.65, 2,169.05)	0.91 (0.78, 0.98)	120.48 (85.04, 168.70)
World Bank Upper Middle Income	12,110.18 (11,292.02, 12,987.79)	6.45 (5.98, 6.85)	671.03 (555.08, 832.94)	4,393.77 (4,115.72, 4,714.37)	1.66 (1.48, 1.82)	259.38 (188.68, 351.68)
World Bank Lower Middle Income	24,340.90 (23,025.33, 25,513.18)	17.16 (14.60, 19.92)	2,143.96 (1,765.05, 2,715.71)	8,420.95 (7,932.39, 8,997.21)	1.66 (1.48, 1.82)	259.38 (188.68, 351.68)
World Bank Low Income	44,320.10 (43,510.02, 45,120.47)	38.30 (31.58, 47.90)	3,170.54 (2,571.98, 3,998.78)	22,224.09 (21,569.43, 22,834.05)	10.45 (8.80, 12.18)	1,206.68 (938.33, 1,540.59)

UI, uncertainty intervals.

At the regional level, the burden of nutritional deficiencies in areas classified with a medium SDI and above continued to decline, with the lowest burden observed in high SDI regions. In low SDI regions, the ASDR and age-standardized DALY rate for nutritional deficiencies witnessed a significant increase between 2010 and 2012, peaking at 36.12 per 100,000 and 3,033.25 per 100,000, respectively; however, a subsequent consistent decreasing trend was observed (Table 1, Figure 1B).

Regionally, although the burden of nutritional deficiencies in Sub-Saharan Africa has diminished relative to 1990, it continues to be higher than in other regions. The ASIR in this region stands at 18,022.35 per 100,000, the ASDR is recorded at 9.56 per 100,000, and the age-standardized DALY rate is measured at 1,120.50 per 100,000. According to the World Bank's regional income statistics, as income levels increase, nutritional deficiencies diminish; the ASIR, ASDR, and age-standardized DALY rates in low-income areas are 11 times, 11 times, and over 10 times higher than those in high-income regions, respectively (Table 1).

Among the 204 countries analyzed, Somalia, Niger, and Chad exhibit the highest ASIR, with values of 83,940.53, 49,780.97, and 36,706.18, respectively. The highest ASDR values are reported in Sierra Leone, South Sudan, and Somalia, at 39.82, 33.90, and 32.07, respectively. The highest age-standardized DALY rates are recorded in Sierra Leone (2,901.99), Mali (2,742.30), and South Sudan (2,426.55). Furthermore, India ranks highest in total incidence, total mortality, and DALYs (Supplementary Tables S1–6, Figures 1C, D).

We further assessed the trends in the SDI across 21 regions worldwide from 1990 to 2021 and explored the potential associations with trends in ASIR, ASDR, and age-standardized DALYs. The results demonstrated that SDI exhibited a significant negative correlation with the ASIR of nutritional deficiencies (correlation coefficient = −0.48, *P* < 0.01), the ASDR (correlation coefficient = −0.51, *P* < 0.01), and the age-standardized DALY rate (correlation coefficient = −0.85, *P* < 0.01) (Figure 1E).

Figure 1F illustrates the results of a subgroup analysis stratified by age. The results revealed that except for high SDI regions, the age-standardized DALY rate for nutritional deficiencies among the < 5 years age group was consistently the highest. Conversely, in high SDI regions, the age-standardized DALY rate was highest among the 70+ years age group.

In 2021, the age-standardized DALY rate attributable to all risk factors associated with nutritional deficiencies was recorded at 611.69 per 100,000. The primary risk factors identified globally and across different SDI regions included iron deficiency, vitamin A deficiency, child underweight, and child wasting. Among these factors, iron deficiency emerged as the predominant risk factor contributing to disabilities and mortality associated with nutritional deficiencies (Figure 1G).

### Global trends in the burden of protein-energy malnutrition

**Figure 2:**
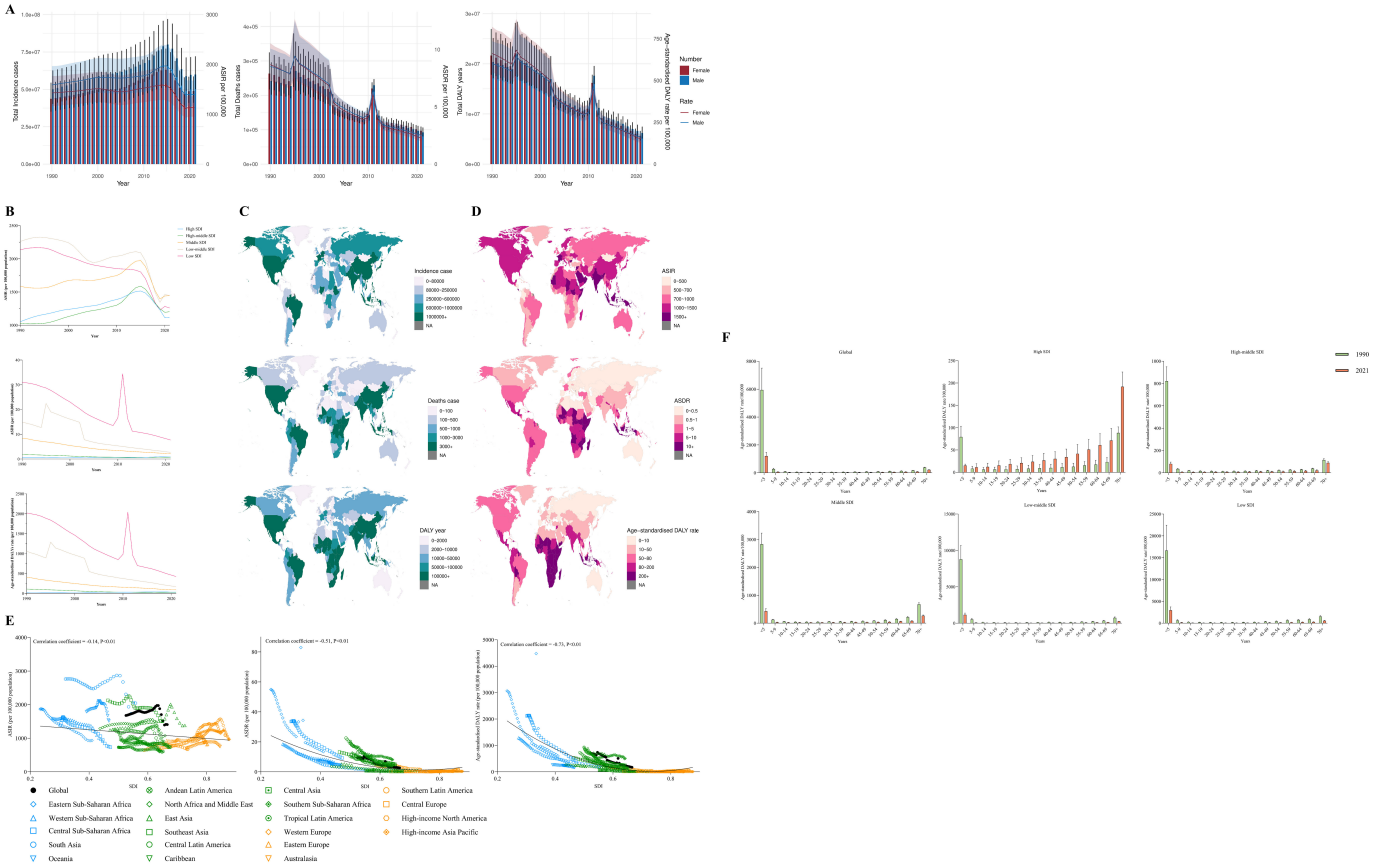
**(A)** The change trends of protrin-energy malnutrition's incidence cases, death cases and DALY from 1990 to 2021. Blue bars represent males and orange bars represent females. **(B)** Trends from 1990 to 2021 in the ASIR, ASDR and Age-standardized DALY rate of protein-energy malnutrition in five SDI regions. **(C, D)** The global disease burden of protein-energy malnutrition in 204 countries or territories. **(E)** The change trends and correlation analyses of ASIR, ASDR and Age-standardized DALY rate with SDI from 1990 to 2021. **(F)** The Age-standardized DALY rate of protein-energy malnutrition in different age groups in 1990 and 2021.

From 1990 to 2021, the global burden of protein-energy malnutrition demonstrated a generally fluctuating decreasing trend. The most substantial reductions were observed in ASDR, which decreased from 9.44 per 100,000 in 1990 to 2.61 per 100,000 in 2021 (with an EAPC of −4.47, indicating a decline of 72.4%), and in the age-standardized DALY rate, which fell from 699.3 per 100,000 in 1990 to 172.38 per 100,000 in 2021 (with an EAPC of −4.64, reflecting a decline of 75.3%). In contrast, the ASIR exhibited considerable fluctuations, with a significant decline occurring primarily after 2014. Furthermore, no significant differences were observed in the ASDR and age-standardized DALY rates for protein-energy malnutrition between males and females; however, the ASIR for males was notably higher than that for females (Table 2, Figure 2A).

**Table 2:** The incidence of protrin-energy malnutrition in 1990/2021.

**Variables**	**1990**			**2021**		
	**ASIR/100, 000 No. (95% UI)**	**ASDR/100, 000 No. (95% UI)**	**Age-standardized DALY rate/100, 000 No. (95% UI)**	**ASIR/100, 000 No. (95% UI)**	**ASDR/100, 000 No. (95% UI)**	**Age-standardized DALY rate/100, 000 No. (95% UI)**
Overall	1,677.02 (1,363.96, 2,070.23)	9.44 (8.16, 11.32)	699.3 (594.81, 869.67)	1,406.21 (1,177.88, 1,691.07)	2.61 (2.29, 2.98)	172.38 (142.75, 204.1)
EAPC-ASIR		−0.20 (−0.52, 0.13)				
EAPC-ASDR		−4.47 (−4.94, −3.99)				
EAPC-Age-standardized DALY rate		−4.64 (−5.11, −4.17)				
**Sex**						
Male	1,766.52 (1,439.35, 2,183.16)	9.49 (8.14, 11.21)	667.59 (564.84, 817.18)	1,549.56 (1,293.16, 1,860.79)	2.77 (2.43, 3.18)	178.38 (146.79, 215)
Female	1,586.98 (1,287.79, 1,964.66)	9.65 (8.07, 11.72)	736.13 (608.81, 912.28)	1,261.43 (1,053.8, 1,509.84)	2.51 (2.21, 2.86)	166.96 (138.67, 196.18)
**SDI**						
High SDI	1,052.68 (828.38, 1,301.59)	0.63 (0.57, 0.68)	21.66 (17.72, 28.31)	1,118.15 (931.89, 1,329.48)	0.88 (0.74, 0.95)	34.33 (25.63, 48.18)
High-middle SDI	1,026.36 (808.06, 1,286.82)	1.81 (1.63, 1.99)	110.29 (98.33, 124.3)	1,202.77 (996.16, 1,464.96)	0.63 (0.55, 0.69)	22.49 (19.18, 26.71)
Middle SDI	1,584.92 (1,292.55, 1,965.1)	8.45 (7.76, 9.03)	402.7 (368.31, 446.62)	1,260.03 (1,043.98, 1,516.13)	7.68 (6.44, 8.91)	424.78 (333.52, 512.5)
Low-middle SDI	1,058.61 (892.98, 1,279.44)	14.96 (12.54, 17.93)	2,260.85 (1,824.5, 2,778.4)	1,447.85 (1,198.87, 1,736.67)	2.74 (2.4, 3.07)	175.06 (148.99, 204.94)
Low SDI	2,134.82 (1,752.88, 2,595.12)	31.07 (25.8, 38.1)	2,019.51 (1,602.83, 2,637.16)	1,445.72 (1,205.61, 1,752.87)	2.17 (1.94, 2.35)	91.34 (79.47, 105.43)
**Region**						
Central Asia	859.35 (650.88, 1,106.48)	0.66 (0.6, 0.72)	66.73 (57.4, 77.58)	741.53 (585.62, 924.03)	0.18 (0.16, 0.21)	12.71 (10.59, 15.29)
East Asia	1,243.43 (992.17, 1,558.93)	5.15 (4.54, 5.75)	241.67 (207.45, 279.94)	1,379.46 (1,129.1, 1,698.95)	0.91 (0.75, 1.06)	17.67 (15.15, 20.36)
South Asia	2,762.86 (2,178.39, 3,458.38)	12.73 (10.14, 15.77)	1,083.59 (891.43, 1,310.04)	2,044.84 (1,655.49, 2,494.51)	1.15 (0.9, 1.44)	165.27 (128.86, 207.35)
Southeast Asia	2,128.86 (1,764.35, 2,623.18)	13.23 (11.16, 15.08)	534.62 (451.28, 654.6)	1,502.74 (1,293.1, 1,744.65)	5.22 (4.44, 5.87)	153.01 (134.56, 173.4)
Central Europe	697.64 (529.02, 886.7)	0.11 (0.1, 0.13)	10.75 (8.87, 13.07)	893.05 (714.88, 1,111.43)	0.32 (0.29, 0.35)	9.7 (8.8, 10.65)
Eastern Europe	934.12 (713.25, 1,182.54)	0.23 (0.21, 0.24)	32.25 (24.89, 40.89)	815.12 (623.72, 1,024.76)	0.15 (0.14, 0.15)	14.1 (10.92, 18.11)
Sub-Saharan Africa	1,604.73 (1,333.11, 1,938.82)	32.42 (27.32, 39.83)	2,029.43 (1,611.67, 2,650.05)	889.44 (740.94, 1,054.76)	9.14 (7.74, 10.61)	451.59 (352.84, 547.78)
Oceania	1,789.31 (1,489.6, 2,115.97)	7.56 (6.28, 9.08)	268.03 (222.04, 322.61)	1,515.6 (1,278.41, 1,752.19)	4.35 (3.56, 5.4)	167.02 (131.17, 209.58)
Latin America and Caribbean	1,147.93 (976.38, 1,375.89)	15.94 (15.13, 16.56)	667.77 (633.36, 706.64)	824.46 (720.82, 947.8)	3.65 (3.25, 4.06)	119.18 (103.42, 139.56)
**World bank income level**						
World bank high income	1,004.88 (789.84, 1,248.63)	0.62 (0.56, 0.66)	23.04 (19.47, 28.86)	1,104.7 (918.77, 1,322.48)	0.83 (0.71, 0.9)	33.42 (25.39, 45.69)
World bank upper middle income	1,138.63 (922.98, 1,418.15)	5.93 (5.51, 6.3)	294.19 (269.6, 321.66)	1,054.21 (883.93, 1,235.64)	9.93 (8.35, 11.56)	512.64 (396.77, 628.22)
World Bank Lower Middle Income	2,246.29 (1,797.05, 2,793.42)	12.94 (10.96, 15.32)	921.1 (774.49, 1,117.99)	1,601.34 (1,318.24, 1,938.5)	2.8 (2.48, 3.14)	177.2 (149.99, 209.86)
World Bank Low Income	1,929.68 (1,633.67, 2,286.46)	36.4 (30.25, 45.35)	2,248.03 (1,764.79, 2,995.97)	1,150.26 (963.98, 1,394.41)	1.45 (1.29, 1.59)	48.56 (43.3, 54.14)

UI, uncertainty intervals.

At the SDI's regional level, the ASIR in middle SDI and higher regions displayed an upward trend before 2014, followed by a marked decline thereafter. By 2021, the ASIR in middle SDI regions was the highest, whereas that in high SDI regions was the lowest. Notably, in low SDI regions, the ASDR and age-standardized DALY rate for protein-energy malnutrition experienced a significant increase from 2010 to 2012, peaking at values of 34.39 per 100,000 and 2,037.19 per 100,000, respectively, before continuing to decline thereafter (Table 2, Figure 2B).

Regionally, in 2021, South Asia reported the highest ASIR for protein-energy malnutrition at 2,044.84 per 100,000, while Sub-Saharan Africa recorded the highest ASDR and age-standardized DALY rates at 9.14 per 100,000 and 451.59 per 100,000, respectively. According to the regional income classifications established by the World Bank, the burden of protein-energy malnutrition has diminished in both the World Bank Lower Middle Income and World Bank Low Income regions compared to 1990. Surprisingly, in 2021, both the ASDR and age-standardized DALY rates for protein-energy malnutrition increased in the World Bank High Income and World Bank Upper Middle-Income regions, while the ASIR in the World Bank High-Income region experienced a slight rise as well (Table 2).

Among the 204 countries, the highest ASIR was reported in the United Arab Emirates (4,688.09), followed by Bulgaria (2,563.81) and India (2,226.25). In contrast, the highest ASDR was observed in Sierra Leone (38.98), South Sudan (32.77), and Somalia (30.89), while the highest age-standardized DALY rates were recorded in Sierra Leone (2,200.17), South Sudan (1,891.77), and Mali (1,533.51). Additionally, India recorded the highest total incidence and total DALY years globally, with 29,505,320.45 cases and 2,035,962.502 years. Meanwhile, Indonesia ranked first in terms of mortality, with 18,148.40 deaths attributed to protein-energy malnutrition (Supplementary Tables S7–12, Figures 2C, D).

Figure 2E illustrates the potential correlation between the trends in changes of the SDI and the ASIR, ASDR, and age-standardized DALYs of protein-energy malnutrition across 21 global regions from 1990 to 2021. The results indicate a significant negative correlation between the SDI and the ASIR of protein-energy malnutrition (correlation coefficient = −0.14, *P* < 0.01), ASDR (correlation coefficient = −0.51, *P* < 0.01), and age-standardized DALY rate (correlation coefficient = −0.73, *P* < 0.01). Figure 2F presents the results of subgroup analyses categorized by age groups. The findings indicate that except for high SDI regions, the age-standardized DALY rate for protein-energy malnutrition in the under-5 age group is consistently the highest. Conversely, in high SDI regions, the age-standardized DALY rate for the 70+ age group is the highest.

### Global trends in the burden of iodine deficiency

**Figure 3:**
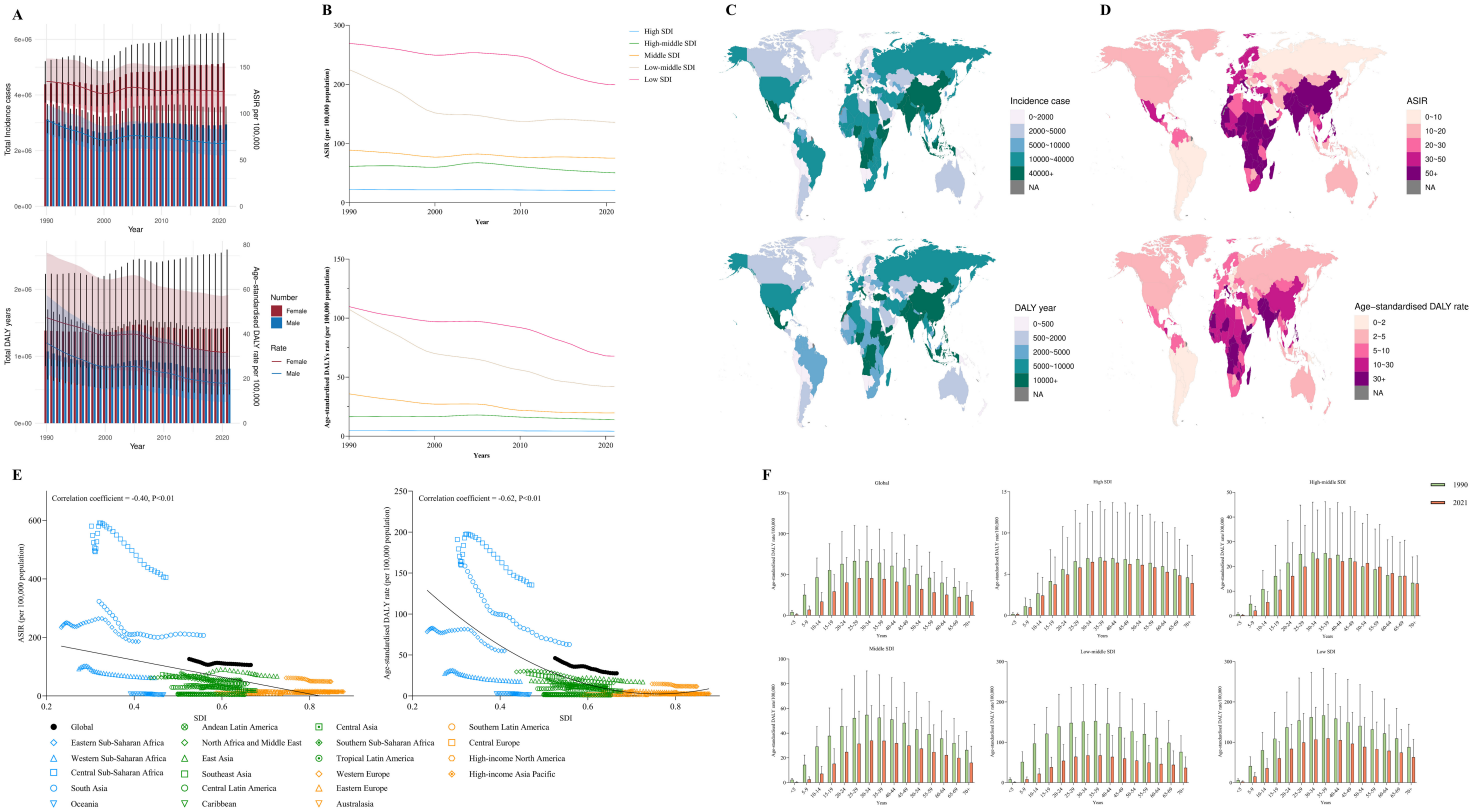
**(A)** The change trends of iodine deficiency's incidence cases and DALY from 1990 to 2021. Blue bars represent males and orange bars represent females. **(B)** Trends from 1990 to 2021 in the ASIR and Age-standardized DALY rate of iodine deficiency in five SDI regions. **(C, D)** The global disease burden of iodine deficiency in 204 countries or territories. **(E)** The change trends and correlation analyses of ASIR and Age-standardized DALY rate with SDI from 1990 to 2021. **(F)** The Age-standardized DALY rate of iodine deficiency in different age groups in 1990 and 2021.

From 1990 to 2021, the global burden of iodine deficiency exhibited a consistent year-on-year decline. The ASIR decreased from 126.14 per 100,000 in 1990 to 105.93 per 100,000 in 2021, reflecting a reduction of 16.2%, with an EAPC of −0.40 (−0.51, −0.29). The decline in the age-standardized DALY rate is even more pronounced, decreasing from 46.19 per 100,000 in 1990 to 27.66 per 100,000 in 2021, representing a decrease of 40.1%, with an EAPC of −1.56 (−1.66, −1.45). Notably, the burden of iodine deficiency is more pronounced in females compared to males (Table 3, Figure 3A).

**Table 3:** The incidence of iodine deficiency in 1990/2021.

**Variables**	**1990**		**2021**	
	**ASIR/100, 000 No. (95% UI)**	**Age-standardized DALY rate/100, 000 No. (95% UI)**	**ASIR/100, 000 No. (95% UI)**	**Age-standardized DALY rate/100, 000 No. (95% UI)**
Overall	126.14 (106.85, 149.86)	46.19 (27.96, 74.29)	105.93 (86.16, 128.28)	27.66 (14.72, 49.47)
EAPC-ASIR	−0.40 (−0.51, −0.29)			
EAPC-Age-standardized DALY rate	−1.56 (−1.66, −1.45)			
**Sex**				
Male	103.28 (87.48, 121.43)	39.98 (24.32, 63.78)	75.49 (61.12, 92.17)	19.98 (10.57, 35.14)
Female	149.69 (127.26, 176.98)	52.52 (32.29, 84.9)	137.72 (112.67, 166.66)	35.43 (18.37, 63.81)
**SDI**
High SDI	22.26 (17.81, 27.15)	4.7 (2.11, 8.93)	20.54 (16.38, 25.01)	4.25 (1.92, 7.95)
High-middle SDI	61.11 (50.64, 73.78)	16.5 (8.6, 29.11)	50.4 (40.83, 61.26)	14.03 (6.93, 25.72)
Middle SDI	88.84 (73.75, 107.29)	35.85 (21.72, 59.08)	75.31 (60.21, 92.44)	19.69 (9.62, 36.81)
Low-middle SDI	224.89 (191.23, 266.24)	107.5 (67.6, 167.85)	135.87 (108.85, 166.15)	41.95 (22.79, 73.82)
Low SDI	269.21 (229.96, 316.2)	269.21 (229.96, 316.2)	199.41 (163.48, 241.93)	67.8 (36.73, 117.79)
**Region**				
Central Asia	27.54 (22.22, 33.79)	9.82 (5.18, 16.52)	18.43 (14.48, 22.35)	5.94 (3.23, 10.01)
East Asia	65.08 (51.67, 81.34)	18.59 (9.98, 33)	67.52 (53.27, 82.61)	17.1 (7.9, 32.75)
South Asia	323.19 (272.79, 385.97)	158.58 (100.23, 246.88)	206.99 (164.96, 255.03)	62.86 (33.98, 112.41)
Southeast Asia	72.99 (59.63, 88.7)	27.77 (16.67, 43.86)	40.62 (31.93, 49.82)	11.28 (6.16, 19.26)
Central Europe	15.55 (12.81, 18.66)	3.72 (1.83, 6.55)	11.48 (9.01, 13.99)	2.38 (1.06, 4.44)
Eastern Europe	11.38 (8.9, 13.94)	4.42 (2.11, 7.29)	11.52 (8.97, 14.06)	4.56 (2.13, 7.59)
Sub-Saharan Africa	200.61 (173.48, 233.79)	63.62 (32.77, 114.28)	151.26 (124.98, 182.69)	44.98 (22.92, 81.28)
Oceania	7.33 (5.77, 9.09)	2.89 (1.65, 4.49)	4.35 (3.44, 5.45)	1.61 (0.9, 2.53)
Latin America and Caribbean	18.72 (15.16, 22.66)	5.47 (2.86, 9.5)	17.63 (14.21, 21.39)	4.8 (2.47, 8.51)
**World Bank Income Level**				
World Bank High Income	31.63 (25.82, 38.22)	7.42 (3.37, 14.08)	25.31 (20.41, 30.62)	5.7 (2.58, 10.79)
World Bank Upper Middle Income	53.66 (43.6, 65.59)	15.64 (8.46, 27.41)	45.9 (37.49, 55.39)	12.99 (6.45, 24.04)
World Bank Lower Middle Income	207.42 (174.84, 246.56)	98.21 (61.96, 153.86)	136.12 (108.61, 167.47)	40.67 (21.99, 72.4)
World Bank Low Income	258.43 (225.3, 300.15)	86.77 (46.37, 153.23)	201.72 (167.83, 242.68)	64.18 (33.73, 113.64)

UI: uncertainty intervals.

At the regional level, the burden of iodine deficiency has decreased to varying degrees across all SDI categories, with more significant reductions observed in low-middle SDI and low SDI regions. Nevertheless, in 2021, the ASIR for iodine deficiency in low SDI regions was 9.71 times higher than that in high SDI regions (Low SDI: 20.54 per 100,000; High SDI: 199.41 per 100,000), while the age-standardized DALY rate was more than 15.95 times higher (Low SDI: 4.25 per 100,000; High SDI: 67.8 per 100,000) (Table 3, Figure 3B).

Regionally, most areas experienced a decline in the burden of iodine deficiency compared to 1990; however, a slight increase was noted in Eastern Europe, with the ASIR rising from 11.38 per 100,000 in 1990 to 11.52 per 100,000 in 2021, and the age-standardized DALY rate increasing from 4.42 per 100,000 in 1990 to 4.56 per 100,000 in 2021. Furthermore, in 2021, the burden of iodine deficiency in South Asia remains the highest, with an ASIR of 206.99 per 100,000 and an age-standardized DALY rate of 62.86 per 100,000.

According to the World Bank's classification of regional income statistics, areas with lower income levels also bear a relatively heavier burden of iodine deficiency (Table 3). Among 204 countries, Somalia, the Democratic Republic of the Congo, and Djibouti rank as the top three in terms of the burden of iodine deficiency, with Somalia's ASIR reaching 725.91 per 100,000. Additionally, India has the highest total number of cases and DALY associated with iodine deficiency globally, with 3,339,819.66 cases and 953,546.19 years (Supplementary Tables S13–16, Figures 3C, D).

Figure 3E illustrates the potential association between the trends of changes in the SDI and the ASIR and age-standardized DALYs of iodine deficiency across 21 global regions from 1990 to 2021. The results indicate a significant negative correlation between SDI and the ASIR of iodine deficiency (correlation coefficient = −0.40, *P* < 0.01) and the age-standardized DALY rate (correlation coefficient = −0.62, *P* < 0.01). Figure 3F presents the results of subgroup analyses based on age groups. The findings demonstrate that the DALYs related to iodine deficiency are primarily concentrated in the population aged over 5 years in both global and various SDI regions. Notably, in comparison to 1990, there was a slight increase in iodine deficiency-related DALYs for the 50–69 age group in high-middle SDI regions in 2021.

### Global trends in the burden of vitamin A deficiency

**Figure 4:**
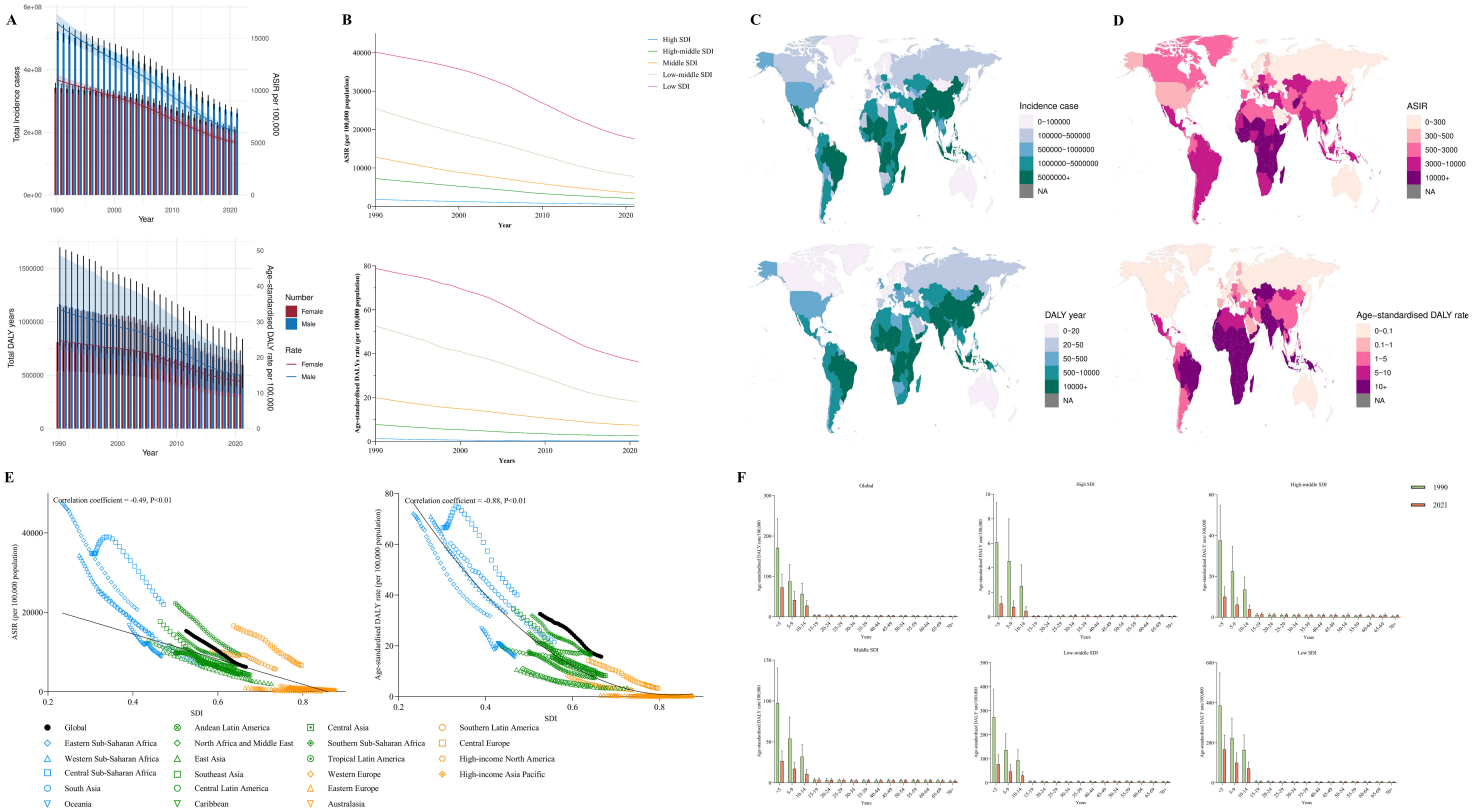
**(A)** The change trends of vitamin A deficiency's incidence cases and DALY from 1990 to 2021. Blue bars represent males and orange bars represent females. **(B)** Trends from 1990 to 2021 in the ASIR and Age-standardized DALY rate of vitamin A deficiency in five SDI regions. **(C, D)** The global disease burden of vitamin A deficiency in 204 countries or territories. **(E)** The change trends and correlation analyses of ASIR and Age-standardized DALY rate with SDI from 1990 to 2021. **(F)** The Age-standardized DALY rate of vitamin A deficiency in different age groups in 1990 and 2021.

From 1990 to 2021, the global burden of vitamin A deficiency demonstrated a consistent downward trend annually. The ASIR decreased from 15,309.38 per 100,000 in 1990 to 6,212.97 per 100,000 in 2021, indicating a reduction of 59.4%, with an EAPC of −2.94 (−3.09, −2.79). The decline in the age-standardized DALY rate was even more pronounced, decreasing from 32.56 per 100,000 in 1990 to 15.73 per 100,000 in 2021, a reduction of 51.7%, with an EAPC of −2.43 (−2.63, −2.24). Furthermore, the burden of vitamin A deficiency is significantly greater in males than in females (Table 4, Figure 4A).

**Table 4:** The incidence of vitamin A deficiency in 1990/2021.

**Variables**	**1990**		**2021**	
	**ASIR/100, 000 No. (95% UI)**	**Age-standardized DALY rate/100, 000 No. (95% UI)**	**ASIR/100, 000 No. (95% UI)**	**Age-standardized DALY rate/100, 000 No. (95% UI)**
Overall	15,309.38 (14,795.26, 15,849.38)	32.56 (21.77, 46.45)	6,212.97 (5,995.41, 6,451.23)	15.73 (10.09, 22.28)
EAPC-ASIR	−2.94 (−3.09, −2.79)			
EAPC-Age-standardized DALY rate	−2.43 (−2.63, −2.24)			
**Sex**				
Male	18,300.34 (17,356.99, 19,260.4)	37.29 (24.94, 54.38)	6,798.94 (6,436.75, 7,190.76)	16.6 (10.58, 23.33)
Female	12,263.74 (11,738.46, 12,824.22)	27.55 (18.35, 38.74)	5,607.26 (5,364.31, 5,864.34)	14.8 (9.59, 21.42)
**SDI**				
High SDI	1,834.56 (1,731.57, 1,950.67)	1.32 (0.84, 1.99)	533.02 (500.63, 567.45)	0.3 (0.19, 0.46)
High-middle SDI	7,251.94 (6,744.03, 7,829.66)	7.78 (5.17, 11.16)	2,052.13 (1,920.15, 2,185.48)	2.72 (1.69, 3.95)
Middle SDI	12,893.56 (12,204.12, 13,560.94)	20.00 (13.18, 28.22)	3,495.26 (3,311.64, 3,685.43)	7.4 (4.83, 10.66)
Low-middle SDI	25,459.19 (24,136.77, 26,659.33)	52.67 (35.03, 75.39)	7,805.61 (7,338.17, 8,319.93)	18.09 (11.61, 26.15)
Low SDI	40,183.53 (39,126.94, 41,255.53)	78.9 (53.42, 113.22)	17,588.14 (17,081.49, 18,160.63)	36.31 (23.71, 51.36)
**Region**				
Central Asia	8,007.29 (7,482.21, 8,568.86)	15.76 (10.21, 24.25)	4,167.24 (3,900.84, 4,475.56)	8.21 (5.32, 11.94)
East Asia	10,226.78 (8,955.07, 11,653.73)	10.66 (6.83, 15.59)	2,003.59 (1,730.21, 2,311.42)	3.45 (2.15, 5.1)
South Asia	25,206.45 (23,113.6, 27,092.84)	60.3 (40.14, 86)	6,920.14 (6,167.34, 7,776.39)	21.49 (13.03, 30.68)
Southeast Asia	17,693.23 (16,665.65, 18,792.08)	34.69 (22.53, 49.8)	4,349.2 (4,016.04, 4,766.59)	8.98 (5.92, 13.27)
Central Europe	16,573.7 (15,835.42, 17,431.9)	14.25 (9, 21.2)	6,575.17 (6,205.13, 6,966)	3.27 (1.99, 4.98)
Eastern Europe	945.74 (869.42, 1025.71)	0.48 (0.28, 0.72)	319.71 (288.44, 351.06)	0.1 (0.06, 0.16)
Sub-Saharan Africa	36,969.54 (36,382.03, 37,522.32)	67.3 (46.15, 94.37)	16,981.65 (16,532.87, 17,387.56)	32.73 (21.6, 46.13)
Oceania	16,646.79 (15,589.96, 17,753.65)	26.67 (17.32, 39.38)	8,809.09 (8,054.27, 9,624.69)	15.62 (9.67, 24.66)
Latin America and Caribbean	15,641.8 (14,739.01, 16,564.8)	17.8 (11.79, 25.3)	6,504.18 (6,048.12, 7,052.52)	7.73 (4.99, 11.1)
**World Bank Income Level**				
World Bank High Income	2,602.21 (2,502.39, 2,718.39)	1.89 (1.2, 2.82)	830.85 (796.64, 868.17)	0.41 (0.26, 0.63)
World Bank Upper Middle Income	10,917.89 (10,124.87, 11,792.92)	12.65 (8.27, 18.17)	3,197.61 (2,984.25, 3,399.66)	4.61 (2.99, 6.56)
World Bank Lower Middle Income	21,887.19 (20,686.2, 22,925.34)	48.66 (32.54, 69.07)	6,683.49 (6,294.25, 7,165.24)	17.99 (11.46, 25.54)
World Bank Low Income	42,131.98 (41,384.63, 42,920.39)	74.32 (50.77, 105.32)	20,968.15 (20,411.02, 21,550.7)	39.51 (26.28, 55.78)

UI, uncertainty intervals.

At the regional level, the burden of vitamin A deficiency has diminished to varying degrees across all SDI categories, with reductions, particularly notable in the low-middle and low SDI regions. Nonetheless, in 2021, the ASIR of vitamin A deficiency in low SDI regions remained 33 times higher than that in high SDI regions (Low SDI: 533.02 per 100,000; High SDI: 17,588.14 per 100,000), while the age-standardized DALY rate was over 121.03 times higher (Low SDI: 0.3 per 100,000; High SDI: 36.31 per 100,000) (Table 4, Figure 4B).

Regionally, in comparison to 1990, the burden of vitamin A deficiency has decreased across all regions. In 2021, the burden of vitamin A deficiency was highest in the Sub-Saharan Africa region, with an ASIR of 16,981.65 per 100,000 and an age-standardized DALY rate of 32.73 per 100,000.

According to regional income statistics from the World Bank, lower-income areas also bear a relatively greater burden of vitamin A deficiency (Table 4). Among 204 countries, Somalia exhibits the highest burden of vitamin A deficiency, with an ASIR of 81,451.76 per 100,000 and an age-standardized DALY rate of 108.60 per 100,000. Additionally, India holds the highest total incidence and DALY years for vitamin A deficiency globally, with 112,700,475 cases and 321,890.3 years (Supplementary Tables S17–20, Figures 4C, D).

Figure 4E illustrates the potential association between the trends of changes in the SDI and the ASIR and age-standardized DALYs of vitamin A deficiency across 21 global regions from 1990 to 2021. The results indicate a significant negative correlation between SDI and the ASIR of vitamin A deficiency (correlation coefficient = −0.49, *P* < 0.01) and the age-standardized DALY rate (correlation coefficient = −0.88, *P* < 0.01). Figure 4F presents the results of subgroup analyses based on age groups. The findings indicate that the DALYs associated with vitamin A deficiency are primarily concentrated in the population aged under 15 years in both global and various SDI regions.

### Global trends in the burden of iron deficiency

**Figure 5:**
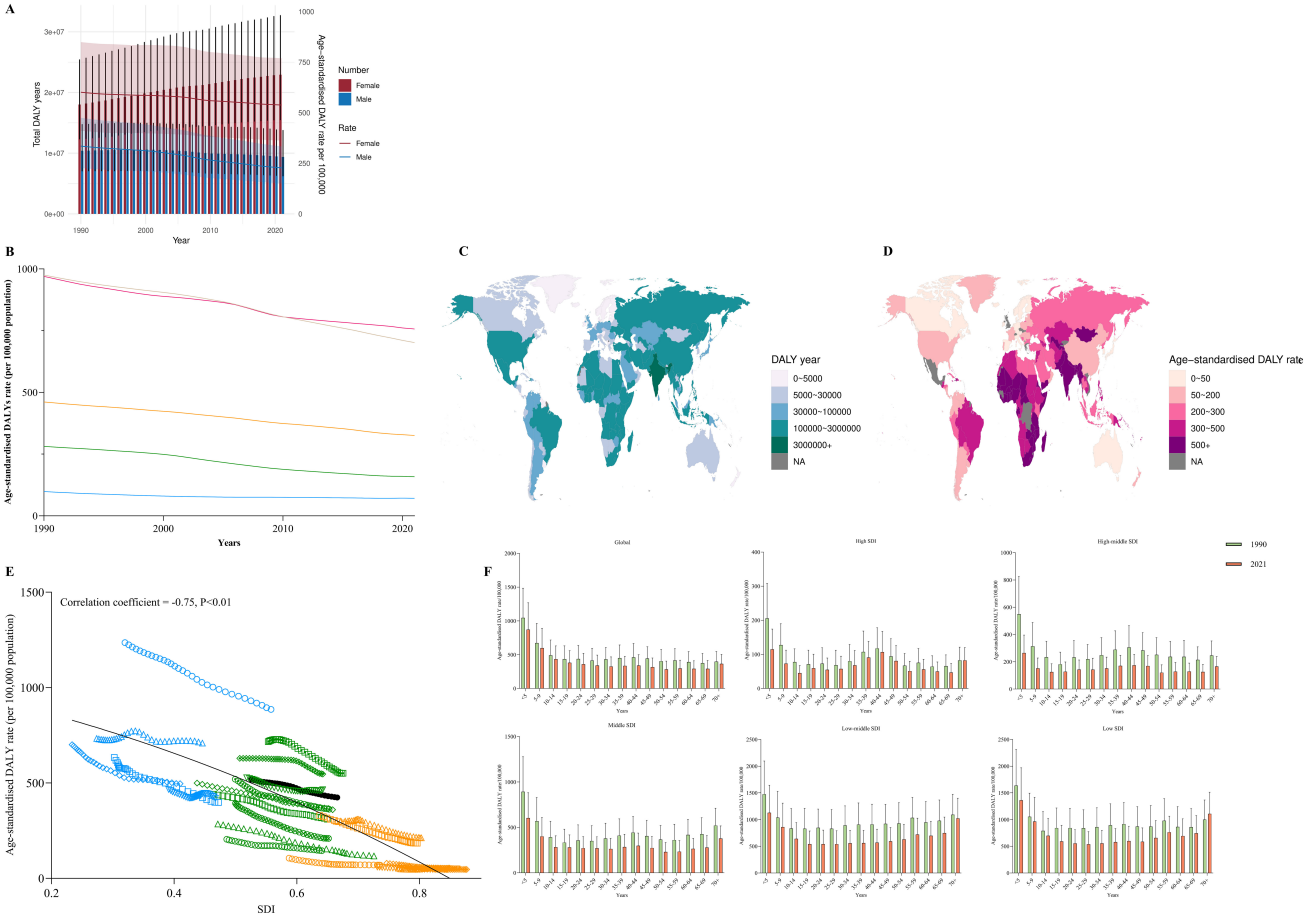
**(A)** The change trends of dietary iron deficiency's DALY from 1990 to 2021. Blue bars represent males and orange bars represent females. **(B)** Trends from 1990 to 2021 in the Age-standardized DALY rate of dietary iron deficiency in five SDI regions. **(C, D)** The global disease burden of dietary iron deficiency in 204 countries or territories. **(E)** The change trends and correlation analyses of Age-standardized DALY rate with SDI from 1990 to 2021. **(F)** The Age-standardized DALY rate of dietary iron deficiency in different age groups in 1990 and 2021.

From 1990 to 2021, the global burden of iron deficiency exhibited a consistent downward trend each year. The age-standardized DALY rate decreased from 517.98 per 100,000 in 1990 to 423.74 per 100,000 in 2021, representing a decline of 18.2%, with an EAPC of −0.68 (−0.72, −0.64). The burden of iron deficiency is significantly greater in females than in males (Table 5, Figure 5A).

**Table 5:** The incidence of dietary iron deficiency in 1990/2021.

**Variables**	**1990**	**2021**
	**Age-standardized DALY rate/100, 000 No. (95% UI)**	**Age-standardized DALY rate/100, 000 No. (95% UI)**
Overall	517.98 (353.99, 730.97)	423.74 (285.27, 610.83)
EAPC-Age-standardized DALY rate	−0.68 (−0.72, −0.64)	
**Sex**		
Male	372.14 (252.28, 528)	253.05 (167.26, 370.92)
Female	668.26 (456.63, 943.34)	597.97 (402.63, 854.44)
**SDI**		
High SDI	98.22 (63.9, 149.04)	71.16 (46.66, 107.1)
High-middle SDI	281.54 (186.61, 410.01)	158.5 (105.24, 230.73)
Middle SDI	461.41 (313.83, 657.08)	325.66 (217.33, 471.71)
Low-middle SDI	975.03 (668.99, 1,368.68)	701.74 (473.82, 995.6)
Low SDI	969.65 (658.73, 1,360.15)	756.7 (507.93, 1,078.97)
**Region**		
Central Asia	716.39 (480.61, 1,034.62)	550.96 (371.78, 796.2)
East Asia	284.97 (192.07, 407.7)	117.23 (77.42, 168.51)
South Asia	1,235.64 (849.62, 1,732.89)	885.49 (600.29, 1,271.36)
Southeast Asia	440.81 (288.92, 632.81)	310.51 (206.2, 450.38)
Central Europe	329.43 (214.17, 480.96)	184.56 (120.76, 272.99)
Eastern Europe	299.72 (196.21, 448.3)	212.2 (143.05, 307.68)
Sub-Saharan Africa	696.16 (473.66, 981.52)	577.35 (385.02, 826.3)
Oceania	466.02 (291.47, 688.36)	419.21 (260.76, 664.25)
Latin America and Caribbean	375.47 (249.35, 533.28)	258.96 (171.4, 370.87)
**World bank income level**		
World bank high income	104.39 (67.43, 157.16)	72.89 (47.5, 109.42)
World bank upper middle income	327.25 (220.53, 469.79)	187.71 (126.46, 270.46)
World bank lower middle income	909.51 (622.89, 1,277.49)	674.98 (456.33, 971.82)
World bank low income	682.28 (464.66, 967.47)	572.49 (385, 816.94)

UI, uncertainty intervals.

At the regional level, although the burden of iron deficiency has diminished to varying degrees across all SDI regions, in 2021, the age-standardized DALY rate in low SDI regions was still more than 10.63 times higher than that in high SDI regions (Low SDI: 71.16 per 100,000; High SDI: 756.7 per 100,000) (Table 5, Figure 5B). Regionally, in comparison to 1990, the burden of iron deficiency has decreased across all regions. In 2021, the burden of iron deficiency was highest in the South Asia region, with an age-standardized DALY rate of 885.49 per 100,000.

According to regional income classifications by the World Bank, the lower-middle income region had the highest age-standardized DALY rate (674.98 per 100,000), whereas the high-income region had the lowest (72.89 per 100,000) (Table 5). Among 204 countries, Yemen exhibited the highest burden of iron deficiency, with an age-standardized DALY rate of 1,405.23 per 100,000. India reported the highest total DALY years attributable to iron deficiency globally, reaching 12,022,677.85 years (Supplementary Tables S21, 22, Figures 5C, D).

Figure 5E illustrates the potential association between the trends of changes in the SDI and age-standardized DALY rates for iron deficiency across 21 global regions from 1990 to 2021. The results indicated a significant negative correlation between SDI and the age-standardized DALY rate for iron deficiency (correlation coefficient = −0.75, *P* < 0.01). Figure 5F presents the results of subgroup analyses based on age groups. The findings indicated that the DALYs associated with iron deficiency were relatively evenly distributed across all age groups globally and within various SDI regions.

## Discussion

This study employed the latest GBD 2021 data to conduct a comprehensive analysis of the disease burden associated with malnutrition and its four subtypes from 1990 to 2021. By calculating the EAPC values for indicators related to the burden of malnutrition over the past 32 years, this study explored the epidemiological trends. The results reveal that significant changes have occurred in the global burden of malnutrition when compared to 1990.

From 1990 to 2021, the global burden of malnutrition and its four subtypes has diminished to varying extents, notably in cases of general malnutrition and vitamin A deficiency. This phenomenon reflects, to a considerable degree, the effective interventions implemented by global public health organizations over the past three decades aimed at promoting disease prevention and control. However, it is concerning that substantial disparities exist in the burden of malnutrition attributable to gender, region, country, socioeconomic status, and age. Firstly, although the global burden of malnutrition exhibits an overall downward trend, significant spatial disparities in disease burden persist across geographical regions, reflecting variances in social and cultural factors. Low-income and low-SDI regions, such as many African countries, continue to bear a substantial burden of disease (30). This situation may be attributed to various factors, including poverty, climate change, educational attainment, and government policies (31). Food security remains a critical global issue, as population growth and increasing consumption continue to drive demand for food. The competition for essential resources such as land, water, and energy further impacts the capacity of nations to produce adequate food supplies (32). A cross-sectional study in Gaza highlighted that chronic food insecurity, exacerbated by war, has led to widespread malnutrition among children, manifesting as stunted growth, wasting, and anemia (33). Similarly, in Kenya, challenges such as limited access to quality seeds, inadequate transportation infrastructure, low value addition, and climate-related food security issues have resulted in nearly 30% of children suffering from nutritional deficiencies, 35% experiencing stunted growth, and approximately 4 million people requiring long-term emergency food assistance (34). Given these challenges, a more effective global strategy is urgently needed to ensure food security and alleviate the burden of nutritional deficiencies. Additionally, infectious diseases play a crucial role in shaping the burden of nutritional deficiencies, creating a vicious cycle that should not be overlooked (35). For instance, nutritional deficiencies have been strongly linked to tuberculosis incidence, disease severity, prognosis, and mortality. Given these associations, further research is needed to explore the mechanisms by which nutritional status influences the effectiveness of tuberculosis vaccines and treatment, particularly in high-burden TB countries (36). Fortunately, countries with low SDI and low-middle SDI have experienced a markedly greater reduction in the burden of malnutrition compared to their counterparts. This is likely primarily due to these countries undergoing a critical period of nutritional transition, coupled with rapid global economic growth and various effective targeted interventions executed by relevant organizations to combat malnutrition (37–39). Some scholars contend that this change signifies that these countries are transitioning to the second phase of the nutritional change model, moving from a decline in famine to the emergence of degenerative diseases; economic growth, urbanization, and technological innovation have rendered obesity and its associated non-communicable diseases increasingly prevalent (40).

Secondly, the global ASIR of protein-energy malnutrition has fluctuated over these 32 years, with even the ASIR in high-middle SDI and high SDI regions witnessing an increase compared to 1990. This shift may be attributed to the dietary habits of populations in these countries gradually transitioning from high-calorie foods to more nutritious alternatives (41). The Mediterranean diet serves as a quintessential example, advocating for the reduction of sugar and red meat intake while promoting the consumption of grains, bread, and legumes to mitigate the risk of cardiovascular diseases (42). However, such a low-calorie dietary pattern may render individuals more susceptible to protein-energy malnutrition. Additionally, it is noteworthy that during the COVID-19 pandemic, the ASIR of protein-energy malnutrition markedly increased in Middle SDI and three lower SDI regions. Previous studies have attributed this phenomenon to the more severe clinical symptoms associated with COVID-19. The inadequacy of prevention and control measures for COVID-19 in lower SDI regions has resulted in many patients being hospitalized, consequently increasing the ASIR of protein-energy malnutrition (43).

Thirdly, the burden of iodine deficiency and dietary iron deficiency is considerably greater among women than men, with a more even age distribution observed for both conditions. This phenomenon may be ascribed to the necessity for pregnant women to provide iodine to their infants, potentially resulting in iodine deficiency for both themselves and their children (44). It is well established that iodine deficiency is linked to a range of diseases, including goiter and hypothyroidism, while adequate thyroid hormone levels are crucial for optimal brain development. Severe iodine deficiency during pregnancy can result in significant neurological and cognitive impairments in children, potentially elevating the risk of infant mortality (45, 46). It is widely acknowledged that during a normal-term pregnancy, a mother requires an intake of 500–800 milligrams of iron (47). It is estimated that the daily dietary iron requirement escalates from 0.8 milligrams per day in early pregnancy to 7.5 milligrams per day in late pregnancy, with an average daily requirement of 4.4 milligrams throughout the pregnancy (48). Consequently, dietary iron deficiency is markedly more prevalent among women than men.

Finally, although the global burden of vitamin A deficiency has markedly decreased over the years, its adverse effects should not be underestimated, particularly in regions with low SDI. The primary causes of vitamin A deficiency encompass insufficient intake of vitamin A-rich foods, inadequate absorption of vitamin A, and losses attributable to various diseases (49). Some scholars posit that pervasive poverty in low SDI regions is a significant factor contributing to the prevalence of vitamin A deficiency, with lower cultural levels further exacerbating the issue (50). A study conducted among the Chinese population revealed that individuals with lower educational attainment exhibit a higher risk of vitamin A deficiency (51). A meta-analysis indicated that in regions characterized by limited income and education levels, enhancing the supply and utilization of vitamin A-rich foods may yield greater benefits than the widespread administration of vitamin A supplements (52).

### Strengths and limitations

In comparison to existing studies, this research possesses several notable advantages. First, leveraging high-quality evidence and data frameworks from the latest GBD 2021 study, we offer a comprehensive analysis of the global trends in the burden of nutritional deficiencies and their four subtypes from 1990 to 2021. This study emphasizes the epidemiological characteristics of nutritional deficiencies across sex, country, region, and age group. Second, our findings provide crucial additional insights into the potential impact of the COVID-19 pandemic on the global burden of nutritional deficiencies, extending, and updating previous research over time.

Nonetheless, this study is not without its limitations. Firstly, the study results are contingent upon aggregated data from the GBD study, with their accuracy reliant on the quality of reporting from various countries. This reliance may lead to a significant number of undiagnosed cases of nutritional deficiencies in certain regions, thereby potentially impacting the accuracy of our findings (53). Secondly, there may be potential data quality issues arising from challenges in accurately determining the number of deaths and DALYs attributable to specific causes, such as nutritional deficiencies.

## Conclusions

From 1990 to 2021, the global burden of nutritional deficiencies has exhibited a general decline. However, in certain regions, particularly low SDI areas, the burden persists at significant levels. Relevant organizations must devise effective, cost-efficient, and targeted interventions tailored to specific regions, genders, ages, and disease subtypes to comprehensively mitigate the adverse impacts of nutritional deficiencies on global public health.

## Data Availability

The original contributions presented in the study are included in the article/Supplementary material, further inquiries can be directed to the corresponding author.

## References

[1] Bal BS Finelli FC Shope TR Koch TR. Nutritional deficiencies after bariatric surgery. Nat Rev Endocrinol. (2012) 8:544–56. 10.1038/nrendo.2012.48 22525731

[2] Christian P Smith ER. Adolescent undernutrition: global burden, physiology, and nutritional risks. Ann Nutr Metab. (2018) 72:316–28. 10.1159/000488865 29730657

[3] Global Food Policy Report 2024: Food Systems for Healthy Diets and Nutrition. Available online at: https://gfpr.ifpri.info/ (accessed September 26, 2024).

[4] Wells JC Sawaya AL Wibaek R Mwangome M Poullas MS Yajnik CS. The double burden of malnutrition: aetiological pathways and consequences for health. Lancet. (2020) 395:75–88. 10.1016/S0140-6736(19)32472-9 31852605 PMC7613491

[5] World Health Organization. Global Nutrition Policy Review: What Does It Take to Scale up Nutrition Action? World Health Organization (2013). Available online at: https://iris.who.int/handle/10665/84408 (accessed September 26, 2024).

[6] Hargreaves D Mates E Menon P Alderman H Devakumar D Fawzi W. Strategies and interventions for healthy adolescent growth, nutrition, and development. Lancet. (2022) 399:198–210. 10.1016/S0140-6736(21)01593-2 34856192

[7] Global Nutrition Report. The state of global nutrition - Global Nutrition Report. Available online at: https://globalnutritionreport.org/reports/2021-global-nutrition-report/ (accessed September 26, 2024).

[8] Kadiyala S Richter L Kulkarni B Chitaya A Harris-Fry H. Global childhood malnutrition. BMJ. (2024) 386:q1874. 10.1136/bmj.q1874 39214530

[9] The Decade of Action on Nutrition 2016-2025 - UNSCN. Available online at: https://www.unscn.org/en/topics/un-decade-of-action-on-nutrition (accessed September 26, 2024).

[10] Savarino G Corsello A Corsello G. Macronutrient balance and micronutrient amounts through growth and development. Italian J Pediatr. (2021) 47:109. 10.1186/s13052-021-01061-0 33964956 PMC8106138

[11] Espinosa Salas S Gonzalez Arias M. Nutrition: Macronutrient Intake, Imbalances, and Interventions. PubMed. (2025). Available online at: https://pubmed.ncbi.nlm.nih.gov/37603617/ (accessed February 8, 2025). 37603617

[12] Golden MH. Proposed recommended nutrient densities for moderately malnourished children. Food Nutr Bull. (2009) 30:S267–342. 10.1177/15648265090303S302 19998863

[13] Nosewicz J Sparks A Hart PA Roberts KM Kaffenberger JA Korman A. The evaluation and management of macronutrient deficiency dermatoses. J Am Acad Dermatol. (2022) 87:640–7. 10.1016/j.jaad.2022.04.007 35427683

[14] Tako E. Dietary Trace Minerals. Nutrients. (2019) 11:2823. 10.3390/nu11112823 31752257 PMC6893782

[15] Zimmermann MB Jooste PL Pandav CS. Iodine-deficiency disorders. Lancet. (2008) 372:1251–62. 10.1016/S0140-6736(08)61005-3 18676011

[16] Timoneda J Rodríguez-Fernández L Zaragozá R Marín M Cabezuelo M Torres L. Vitamin A deficiency and the lung. Nutrients. (2018) 10:1132. 10.3390/nu10091132 30134568 PMC6164133

[17] Bayati M Arkia E Emadi M. Socio-economic inequality in the nutritional deficiencies among the world countries: evidence from global burden of disease study 2019. J Health, Popul Nutr. (2025) 44:8. 10.1186/s41043-025-00739-z 39806471 PMC11731139

[18] Chong B Jayabaskaran J Kong G Chan YH Chin YH Goh R. Trends and predictions of malnutrition and obesity in 204 countries and territories: an analysis of the Global Burden of Disease Study 2019. EClinicalMedicine. (2023) 57:101850. 10.1016/j.eclinm.2023.101850 36864983 PMC9971264

[19] Silverio R Gonçalves DC Andrade MF Seelaender M. Coronavirus Disease 2019 (COVID-19) and nutritional status: the missing link? Adv Nutr. (2021) 12:682–92. 10.1093/advances/nmaa125 32975565 PMC7543263

[20] Zhou L Deng Y Li N Zheng Y Tian T Zhai Z. Global, regional, and national burden of Hodgkin lymphoma from 1990 to 2017: estimates from the 2017 Global Burden of Disease study. J Hematol Oncol. (2019) 12:107. 10.1186/s13045-019-0799-1 31640759 PMC6805485

[21] Rezaei F Mazidimoradi A Rayatinejad A Allahqoli L Salehiniya H. Temporal trends of tracheal, bronchus, and lung cancer between 2010 and 2019, in Asian countries by geographical region and sociodemographic index, comparison with global data. Thoracic Cancer. (2023) 14:1668–706. 10.1111/1759-7714.14912 37127553 PMC10290923

[22] GBD 2019 Diseases and Injuries Collaborators. Global burden of 369 diseases and injuries in 204 countries and territories, 1990-2019: a systematic analysis for the Global Burden of Disease Study 2019. Lancet. (2020) 396:1204–1222. 10.1016/S0140-6736(20)30925-9 33069326 PMC7567026

[23] Hua Z Wang S Yuan X. Trends in age-standardized incidence rates of depression in adolescents aged 10–24 in 204 countries and regions from 1990 to 2019. J Affect Disord. (2024) 350:831–7. 10.1016/j.jad.2024.01.009 38242215

[24] GBD 2017 Inflammatory Bowel Disease Collaborators. The global, regional, and national burden of inflammatory bowel disease in 195 countries and territories, 1990–2017: a systematic analysis for the Global Burden of Disease Study 2017. Lancet Gastroenterol Hepatol. (2020) 5:17. 10.1016/S2468-1253(19)30333-4 31648971 PMC7026709

[25] GBD 2017 Causes of Death Collaborators. Global, regional, and national age-sex-specific mortality for 282 causes of death in 195 countries and territories, 1980-2017: a systematic analysis for the Global Burden of Disease Study 2017. Lancet. (2018) 392:1736–1788. 10.1016/S0140-6736(18)32203-7 30496103 PMC6227606

[26] Lv B Lan J-X Si Y-F Ren Y-F Li M-Y Guo F-F. Epidemiological trends of subarachnoid hemorrhage at global, regional, and national level: a trend analysis study from 1990 to 2021. Military Med Res. (2024) 11:46. 10.1186/s40779-024-00551-6 38992778 PMC11241879

[27] Li X Yang C Lv J Liu H Zhang L Yin M. Global, regional, and national epidemiology of migraine and tension-type headache in youths and young adults aged 15–39 years from 1990 to 2019: findings from the global burden of disease study 2019. J Headache Pain. (2023) 24:126. 10.1186/s10194-023-01659-1 37718436 PMC10506184

[28] Global Burden of Disease Study 2019 (GBD 2019) Cause List Mapped to ICD Codes | GHDx. Available online at: https://ghdx.healthdata.org/record/ihme-data/gbd-2019-cause-icd-code-mappings (accessed September 26, 2024).

[29] Lv B Song G Jing F Li M Zhou H Li W. Mortality from cerebrovascular diseases in China: exploration of recent and future trends. Chinese Med J. (2023) 137:588–95. 10.1097/CM9.0000000000002760 37415525 PMC10932538

[30] Institute for Health Metrics and Evaluation. Available online at: https://www.healthdata.org/taxonomy/glossary/socio-demographic-index-sdi#:%7E:text=A%20summary%20measure%20that%20identifies,areas%20in%20the%20GBD%20study (accessed September 28, 2024).

[31] WHO Expert Consultation. Appropriate body-mass index for Asian populations and its implications for policy and intervention strategies. Lancet. (2004) 363:157–163. 10.1016/S0140-6736(03)15268-3 14726171

[32] Godfray HCJ Beddington JR Crute IR Haddad L Lawrence D Muir JF. Food security: the challenge of feeding 9 billion people. Science. (2010) 327:812–8. 10.1126/science.1185383 20110467

[33] Horino M Zaqqout R Habash R Albaik S Abed Y Al-Jadba G. Food insecurity, dietary inadequacy, and malnutrition in the Gaza Strip: a cross-sectional nutritional assessment of refugee children entering the first grade of UNRWA schools and their households before the conflict of 2023–24. Lancet Global Health. (2024) 12:e1871–80. 10.1016/S2214-109X(24)00320-6 39326434

[34] Kimiywe J. Food and nutrition security: challenges of post-harvest handling in Kenya. Proc Nutr Soc. (2015) 74:487–95. 10.1017/S0029665115002414 26260147

[35] Sinha P Guerrant RL. The costly vicious cycle of infections and malnutrition. J Infect Dis. (2023) 229:1611–3. 10.1093/infdis/jiad513 37972258 PMC11175688

[36] Sinha P Lönnroth K Bhargava A Heysell SK Sarkar S Salgame P. Food for thought: addressing undernutrition to end tuberculosis. Lancet Infect Dis. (2021) 21:e318–25. 10.1016/S1473-3099(20)30792-1 33770535 PMC8458477

[37] O'Connell SA Smith C. Economic growth and child undernutrition. Lancet Global Health. (2016) 4:e901–2. 10.1016/S2214-109X(16)30250-9 27855863

[38] Vollmer S Harttgen K Subramanyam MA Finlay J Klasen S Subramanian SV. Association between economic growth and early childhood undernutrition: evidence from 121 Demographic and Health Surveys from 36 low-income and middle-income countries. Lancet Global Health. (2014) 2:e225–34. 10.1016/S2214-109X(14)70025-7 25103063

[39] Gillespie S Haddad L Mannar V Menon P Nisbett N. The politics of reducing malnutrition: building commitment and accelerating progress. Lancet. (2013) 382:552–69. 10.1016/S0140-6736(13)60842-9 23746781

[40] Popkin BM. An overview on the nutrition transition and its health implications: the Bellagio meeting. Public Health Nutr. (2002) 5:93–103. 10.1079/PHN2001280 12027297

[41] Goh EV Azam-Ali S McCullough F Roy Mitra S. The nutrition transition in Malaysia; key drivers and recommendations for improved health outcomes. BMC Nutr. (2020) 6:32. 10.1186/s40795-020-00348-5 32612845 PMC7322903

[42] Berry EM. Sustainable food systems and the mediterranean diet. Nutrients. (2019) 11:2229. 10.3390/nu11092229 31527411 PMC6769950

[43] Fedele D De Francesco A Riso S Collo A. Obesity, malnutrition, and trace element deficiency in the coronavirus disease (COVID-19) pandemic: an overview. Nutrition. (2021) 81:111016. 10.1016/j.nut.2020.111016 33059127 PMC7832575

[44] Pearce EN Lazarus JH Moreno-Reyes R Zimmermann MB. Consequences of iodine deficiency and excess in pregnant women: an overview of current knowns and unknowns. Am J Clin Nutr. (2016) 104:918S−23S. 10.3945/ajcn.115.110429 27534632 PMC5004501

[45] Zimmermann MB. Iodine deficiency. Endocr Rev. (2009) 30:376–408. 10.1210/er.2009-0011 19460960

[46] de Escobar GM Obregón MJ del Rey FE. Iodine deficiency and brain development in the first half of pregnancy. Public Health Nutr. (2007) 10:1554–1570. 10.1017/S1368980007360928 18053280

[47] Milman N Bergholt T Byg K-E Eriksen L Graudal N. Iron status and iron balance during pregnancy. A critical reappraisal of iron supplementation. Acta Obstetr Gynecol Scandinavica. (1999) 78:749–57. 10.1080/j.1600-0412.1999.780902.x 10535335

[48] Milman N. Prepartum anaemia: prevention and treatment. Ann Hematol. (2008) 87:949–59. 10.1007/s00277-008-0518-4 18641987

[49] Global prevalence of vitamin A deficiency in populations at risk 1995-2005 : WHO global database on vitamin A deficiency. Available online at: https://www.who.int/publications/i/item/9789241598019 (accessed September 28, 2024).

[50] Black RE Victora CG Walker SP Bhutta ZA Christian P de Onis M. Maternal and child undernutrition and overweight in low-income and middle-income countries. Lancet. (2013) 382:427–51. 10.1016/S0140-6736(13)60937-X 23746772

[51] Yang C Chen J Liu Z Yun C Piao J Yang X. Prevalence and influence factors of vitamin A deficiency of Chinese pregnant women. Nutr J. (2015) 15:12. 10.1186/s12937-016-0131-7 26818747 PMC4729160

[52] Bassey C Crooks H Paterson K Ball R Howell K Humphries-Cuff I. Impact of home food production on nutritional blindness, stunting, wasting, underweight and mortality in children: a systematic review and meta-analysis of controlled trials. Crit Rev Food Sci Nutr. (2020) 62:1856–69. 10.1080/10408398.2020.1848786 33272038

[53] Yi M Li A Zhou L Chu Q Song Y Wu K. The global burden and attributable risk factor analysis of acute myeloid leukemia in 195 countries and territories from 1990 to 2017: estimates based on the global burden of disease study 2017. J Hematol Oncol. (2020) 13:72. 10.1186/s13045-020-00908-z 32513227 PMC7282046

