# Supplementary figures and images for: Global, regional, and national burden of nutritional deficiencies spanning from 1990 to 2021, with a focus on the impacts observed during the COVID-19 pandemic
Source: Front Nutr. 2025 May 8;12:1535566. doi: 10.3389/fnut.2025.1535566 (PMC12094969; doi:10.3389/fnut.2025.1535566)

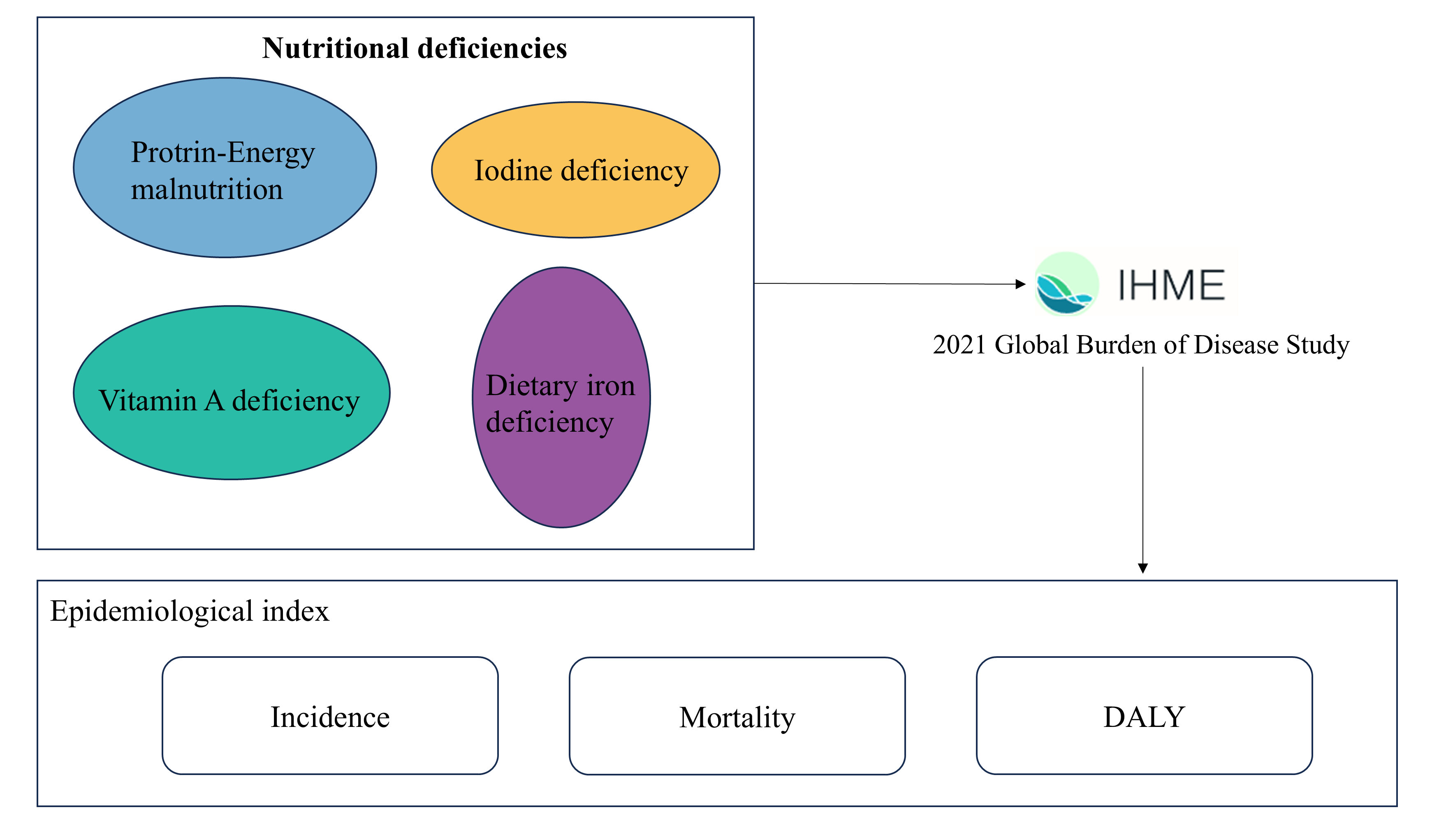

Supplement: Supplementary file 1 [file Image_1.tif]
